# Variable responses of human microbiomes to dietary supplementation with resistant starch

**DOI:** 10.1186/s40168-016-0178-x

**Published:** 2016-06-29

**Authors:** A. Venkataraman, J. R. Sieber, A. W. Schmidt, C. Waldron, K. R. Theis, T. M. Schmidt

**Affiliations:** Department of Internal Medicine, University of Michigan, Ann Arbor, MI 48105 USA; Present address: Department of Biology, University of Minnesota, Duluth, MN 55812 USA; Present address: Department of Immunology and Microbiology, Wayne State University School of Medicine, Detroit, MI 48201 USA

## Abstract

**Background:**

The fermentation of dietary fiber to various organic acids is a beneficial function provided by the microbiota in the human large intestine. In particular, butyric acid contributes to host health by facilitating maintenance of epithelial integrity, regulating inflammation, and influencing gene expression in colonocytes. We sought to increase the concentration of butyrate in 20 healthy young adults through dietary supplementation with resistant starch (unmodified potato starch—resistant starch (RS) type 2).

**Methods:**

Fecal samples were collected from individuals to characterize butyrate concentration via liquid chromatography and composition of the microbiota via surveys of 16S rRNA-encoding gene sequences from the Illumina MiSeq platform. Random Forest and LEfSe analyses were used to associate responses in butyrate production to features of the microbiota.

**Results:**

RS supplementation increased fecal butyrate concentrations in this cohort from 8 to 12 mmol/kg wet feces, but responses varied widely between individuals. Individuals could be categorized into three groups based upon butyrate concentrations before and during RS: enhanced, high, and low (*n* = 11, 3, and 6, respectively). Fecal butyrate increased by 67 % in the enhanced group (from 9 to 15 mmol/kg), while it remained ≥11 mmol/kg in the high group and ≤8 mmol/kg in the low group. Microbiota analyses revealed that the relative abundance of RS-degrading organisms—*Bifidobacterium adolescentis* or *Ruminococcus bromii*—increased from ~2 to 9 % in the enhanced and high groups, but remained at ~1.5 % in the low group. The lack of increase in RS-degrading bacteria in the low group may explain why there was no increase in fecal butyrate in response to RS. The microbiota of individuals in the high group were characterized by an elevated abundance of the butyrogenic microbe *Eubacterium rectale* (~6 % in high *vs.* 3 % in enhanced and low groups) throughout the study.

**Conclusions:**

We document the heterogeneous responses in butyrate concentrations upon RS supplementation and identify characteristic of the microbiota that appear to underlie this variation. This study complements and extends other studies that call for personalized approaches to manage beneficial functions provided by gut microbiomes.

## Background

The microbiota in the large intestine provides several functions that are beneficial to human health such as producing short-chain fatty acids, modifying primary to secondary bile acids, and providing colonization resistance to some enteric pathogens [1, 2]. Managing this community of microbes to maintain and improve these beneficial functions could promote health and reduce the incidence of preventable diseases including obesity and type 2 diabetes [3], colon cancer [4], chronic and acute undernutrition [5], and infections by *Clostridium difficile* [6].

One of the beneficial functions derived from the colonic microbiota is the production of butyric acid that is generated from the fermentation of dietary fiber. The conjugate base of the acid—butyrate—is the preferred energy source for colonocytes [7]. Butyrate improves the intestinal barrier by facilitating tight-junction assembly [8], suppresses inflammatory and allergic responses by inducing differentiation of colonic regulatory T cells [9], regulates cell apoptosis [10], and stimulates production of anorectic hormones [11]. Indeed, reduced concentrations of butyrate have been associated with the incidence of graft-versus-host disease [12, 13], kwashiorkor [14], colon cancer [15], and obesity [11]. Especially in these circumstances, increased butyrate production in the large intestine may be beneficial to human health.

One approach to increasing butyrate production is to deliver more fermentable carbohydrates to gut microbiomes. Here, we report on the impact of dietary supplementation with resistant starch (unmodified potato starch; RS type 2) on fecal butyrate concentrations and composition of the gut microbiota in 20 healthy young adults. RS consists of starch that is resistant to hydrolysis by human enzymes and passes through the small intestine unabsorbed. In the large intestine, RS can be metabolized and then fermented by microbes to a variety of products, including butyric acid [16]. In this study, we find that the response to RS supplementation varies between individuals in ways that can be explained, at least in part, by variation in the composition of their microbiota. Recognizing inter-individual variability in responses to fiber supplementation and determining the microbiota characteristics that underlie it are essential first steps towards personalized plans for managing gut microbiomes for desirable functionality, including the production of butyrate.

## Methods

### Participants

Prospective volunteers were students in research-based sections of Introductory Biology 173 at the University of Michigan. Individuals with a self-reported history of bowel disorders such as irritable bowel syndrome, inflammatory bowel disease, or colorectal cancer were excluded from the study. Twenty individuals (10 males, 10 females) participated in the 3-week study. The age range was 19–20 years, and the BMI range was from 19–63. Each participant gave his or her written, informed consent before participating in the study. This research was carried out in compliance with the Helsinki Declaration and was approved by the Institutional Review Board of the University of Michigan Medical School (HUM00094242).

### Study design

**Fig. 1:**
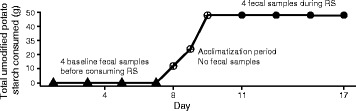
Experimental design. Participants supplemented their habitual diets with resistant starch. Filled symbols represent points at which fecal samples were collected

Participants consumed their habitual diet throughout the study period. During the intervention phase, raw unmodified potato starch (Bob’s Red Mill, Milwaukie, OR) was gradually added to their diet (day 1—12 g, day 2—24 g, day 3—48 g; Fig. 1). This potato starch contains approximately 50 % resistant starch (type 2) by weight. After the acclimatization period, the subjects consumed 48 g of potato starch—24-g doses twice per day—for seven more days. Participants were provided a 1-tablespoon scoop, which they used to measure out the appropriate amount of potato starch (each tablespoon ≈12 g) and consumed it after mixing the starch with cold water.

### Fecal collection

Participants were provided waxed tissue paper (Epitope Diagnostics, San Diego, CA), which was laid down on the water in the toilet bowl prior to defecation. This paper sticks to the sides of the toilet bowl so that the fecal sample is not readily contaminated by water in the toilet. Fecal samples were collected using the deoxyribose nucleic acid (DNA) Genotek Omnigene Gut Collection kits (DNA Genotek, Ontario, Canada) following the manufacturer’s instructions. The DNA Genotek tube contains 2 mL of a stabilization buffer and a steel ball to facilitate mixing of the fecal material with the buffer. Approximately, 0.67 g of fecal material was collected per tube. The kits were returned to a −20 °C freezer within 24 h of collection. Frozen samples were thawed and aliquots were withdrawn for DNA extraction, measurement of fermentation products, and to create sample archives.

### DNA extraction, PCR, sequencing, and sequence processing

For DNA extraction, 0.25 mL of the fecal suspension in the DNA Genotek tube was deposited into a MoBio PowerMag Soil DNA Isolation Bead Plate. DNA was extracted following MoBio’s instructions on an epMotion 5075 liquid handling workstation (Eppendorf, Hauppauge, NY). The V4 region of the 16S-ribosomal RNA (rRNA) encoding genes was amplified from each sample using the dual-indexing sequencing strategy [17]. Sequencing was performed on the Illumina MiSeq platform, using a MiSeq Reagent Kit V2 500 cycles (Cat# MS-102-2003), according to the manufacturer’s instructions. Sequences were curated using mothur v1.31.2 [18] and clustered into operational taxonomic units at ≥ 97 % sequence identity using the average neighbor algorithm.

### Measurement of fermentation products

One millimeter of fecal suspension was aliquoted from each DNA Genotek OmniGut tube and centrifuged at 4 °C for 10 min (4500×*g*). The supernatant fraction was withdrawn and passed sequentially through 1.2, 0.65, and 0.22-μm hydrophilic low protein-binding Durapore membrane filters (EMD Millipore, Darmstadt, Germany). Filtered samples were stored at −20 °C until analysis. Once thawed, samples were maintained at 4 °C in an autosampler. A Shimadzu HPLC (Shimadzu Scientific Instruments, Columbia, MD) equipped with a dual UV (214 nm for short-chain fatty acids (SCFAs))/refractive index detector (for ethanol) and an Aminex HPX-87H column (Bio-Rad Laboratories, Hercules, CA) heated to 50 °C was used to measure acetic, propionic, and butyric acids and ethanol in the filtrate. The mobile phase was 0.01 N H_2_SO_4_ at a flow rate of 0.6 ml/min, and the injection volume was 10 μl. Samples were randomized in regard to injection order, and the average value from two technical replicates was used in all subsequent analyses. Ethanol, which was a component of the stabilization buffer, served to monitor injection volumes. Samples in which the peak height for ethanol was less than 70 % of the average peak height for ethanol in all samples were excluded from further analysis. Eight external standards (0.1–20 mM) for acetic, propionic, and butyric acids were used to generate the standard curve. These standards were run after every 100 samples. LC Solutions Software (Shimadzu Scientific Instruments, Columbia, MD) was used to curate the data and calculate concentrations based on the standard curve generated during the run. The concentrations were then normalized by the average weight of the fecal samples (0.67 g) collected in the DNA Genotek tubes.

### Statistical analyses

All statistical analyses were performed using RStudio version 0.99.489 [19] and the software PAST [20]. To evaluate the impact of RS supplementation on acetate, propionate, and butyrate concentrations in the study population, a nested repeated measures analysis of variance (ANOVA) was used. This revealed that fecal butyrate and acetate concentrations increased with RS intake in the study population (*p* < 0.05). For all subsequent analyses, the median value of butyrate for each person before and during RS consumption was used so that the number of samples compared was not artificially inflated. Paired or unpaired *t* tests were employed as appropriate. To determine if RS intake altered microbial community composition, a permutational analysis of variance (PERMANOVA) analysis was conducted with the Bray-Curtis similarity index. Each individual was used as the blocking factor to account for repeated measures of microbiota composition from each individual. This analysis revealed that RS intake altered the composition of gut microbiota in the study population (*p* < 0.05). For further analyses, we used the median abundance of each operational taxonomic unit (OTU) in each individual before and during RS consumption. To identify specific OTUs that had changed with RS consumption, LEfSe and Random Forest analyses were performed for each individual separately (comparing their four samples before and four samples during RS consumption). LEfSe [21] was implemented within mothur using the correction for multiple comparisons. PERMANOVA, analysis of similarity (ANOSIM), and permutational analyses of dispersions (PERMDISP) analyses were performed with the package vegan in R. Random Forest was implemented using the package randomForest in R.

## Results

The impact of RS on the composition of the gut microbiota and the concentration of three short-chain fatty acids (acetic, propionic, and butyric) in feces was determined in 20 healthy young adults. RS was gradually introduced into their regular diets during an acclimatization period of 3 days (Fig. 1). Thereafter, study participants included 48 g of unmodified potato starch in their daily diet (~24 g as RS) for 7 days. Four fecal samples were collected before the introduction of RS and another four during the period of maximum RS supplementation (Fig. 1).

There was considerable intra-individual variability in the concentration of acetate, propionate, and butyrate (coefficient of variation = 20–90 %). This variability is not unexpected because fecal SCFA concentrations are influenced by host absorption, transit time through the GI tract, host diet, and time since last meal. Due to this variability and the number of samples, Student’s *t* tests did not reveal measurable differences in the concentration of SCFA in response to RS supplementation for an individual (four samples before *vs.* four during RS for each individual; *p* ≥ 0.10 for all individuals). However, a repeated measures ANOVA showed that RS supplementation increased the fecal concentration of butyrate by 50 % and acetate by 26 % in the study population as a whole (*p* = 0.02 and 0.03, respectively; Table 1). The concentrations of propionate did not change significantly in this cohort (*p* = 0.85; Table 1).

**Table 1:** Effect of dietary supplementation with RS on concentration of select fermentation products (mmol/kg wet feces)

Fermentation product	Before RS	During RS	Change	*p* value (repeated measures ANOVA)
	Median ± IQR	Median ± IQR		
Butyric acid	8 ± 6	12 ± 7	50 %	0.03
Acetic acid	27 ± 6	34 ± 10	26 %	0.02
Propionic acid	13 ± 6	12 ± 5	−8 %	0.85

*IQR* interquartile range

Like the fecal SCFA concentrations, there was also some intra-individual variability in the composition of microbiota. However, at least 75 % of OTUs were consistently detected in all samples from an individual with the coefficient of variation in their relative abundances ranging from 39 to 65 %. Unlike the SCFAs, where statistical tests did not detect differences in response to RS for an individual, ANOSIM tests showed that RS supplementation altered the composition of microbiota in most individuals in the study (ANOSIM with Bray-Curtis similarity; four samples before *vs.* four during RS for each individual; *p* < 0.10 for 16 out of 20 individuals). This conclusion was corroborated by results from a PERMANOVA analysis to determine if RS supplementation altered the composition of microbiota in the overall study population. In order to incorporate intra-individual variability, each individual was considered as the blocking factor in the PERMANOVA. This analysis revealed that the composition of the gut microbiota was altered with RS supplementation in our study population (PERMANOVA using Bray-Curtis similarity blocking for each individual; *p* = 0.001). A PERMDISP analysis further revealed that the PERMANOVA was not affected by differences in the dispersion of communities before and during RS (PERMDISP *p* = 0.44).

The relative abundance of OTUs belonging to the phylum *Actinobacteria* increased with RS, and there was a small decrease in the abundance of *Firmicutes*. No changes were detected in the relative abundances of *Bacteroidetes* or *Proteobacteria* (Table 2). Finally, RS supplementation did not change the overall richness or evenness of the microbial community in the study population (repeated measures ANOVA Chao1 index before *vs.* during *p* = 0.49; Simpson’s index before *vs.* during *p* = 0.96).

**Table 2:** Effect of dietary supplementation with RS on relative abundance of four dominant bacterial phyla

Phylum	Before RS	During RS (%)	Change	*p* value
	Median ± IQR	Median ± IQR		paired *t* test
*Actinobacteria*	1.3 ± 0.6	6.2 ± 0.6	377 %	0.02
*Firmicutes*	37.1 ± 10.7	33.2 ± 5.2	-11 %	0.04
*Bacteroidetes*	53.3 ± 13.2	51.5 ± 9	-3 %	0.82
*Proteobacteria*	4.7 ± 4.1	4.8 ± 4.1	2 %	0.82

*IQR* interquartile range

**Fig. 2:**
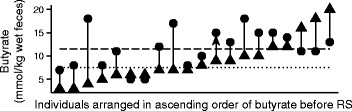
Median butyrate concentrations for each individual before (*triangles*) and during consumption of RS (*circles*). *Dotted* and *dashed lines* denote the median values for butyrate before and during RS, respectively, for the entire study population

Therefore, despite the intra-individual variability in fecal SCFA concentrations and composition of microbiota, repeated measures ANOVA and PERMANOVA reveal that RS consumption led to increases in fecal butyrate concentrations and altered the composition of the microbiota in the study population. However, the starting concentrations of butyrate and the changes in butyrate during RS varied widely between individuals (Fig. 2). Baseline butyrate concentrations were not predictive of butyrate concentrations during RS supplementation (*R*^2^ = 0.08, *p* = 0.20).

To identify characteristics of the microbiota that may underlie the variable responses to RS, we first used Random Forest regression to identify relationships between the abundances of OTUs and butyrate concentrations before and during consumption of RS. No OTUs were particularly strong predictors of butyrate concentrations either before or during RS consumption. Butyrate concentrations before RS were weakly related to baseline abundances of OTU 4 (*Eubacterium rectale*) (*R*^2^ = 0.14; *p* = 0.10). Unexpectedly, this relationship was not detectable during RS supplementation.

**Fig. 3:**
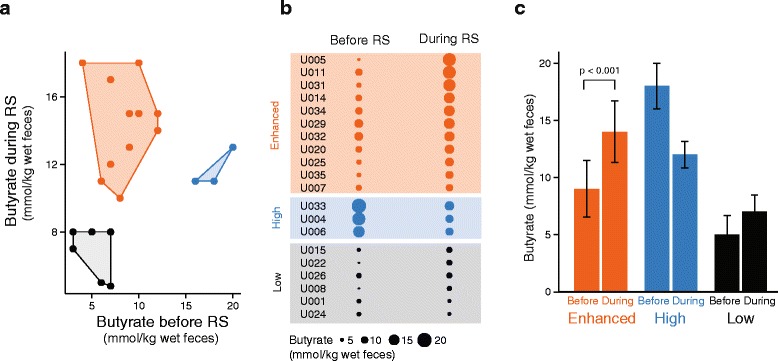
Clustering individuals based upon their butyrate response to RS. **a** Three groups generated by k-means clustering. **b** Median butyrate concentrations before and during RS supplementation. **c** Average butyrate concentrations before and during RS in the three groups

Population-wide relationships between OTUs and butyrate concentrations could be masked by the heterogeneity of both variables between individuals. We therefore looked for correlations between features of the microbiota and butyrate concentrations in subsets of participants that had similar responses in fecal butyrate following RS supplementation. The study population was separated into three groups using k-means clustering based on butyrate concentrations before and during RS. An elbow plot [22] revealed that there were three “clusters”. The categories identified were enhanced, high, and low (Fig. 3a, b). The concentration of butyrate in the “enhanced” group (*n* = 11) increased significantly following consumption of RS (from 9 to 15 mmol/kg wet feces, paired *t* test *p* = 0.0003). Individuals in the “high” group (*n* = 3) maintained butyrate concentrations ≥11 mmol/kg wet feces during the course of the study. Individuals in the “low” group (*n* = 6) had less than or equal to 8 mmol butyrate/kg wet feces both before and during RS (paired *t* test *p* = 0.14; Fig. 3c).

**Fig. 4:**
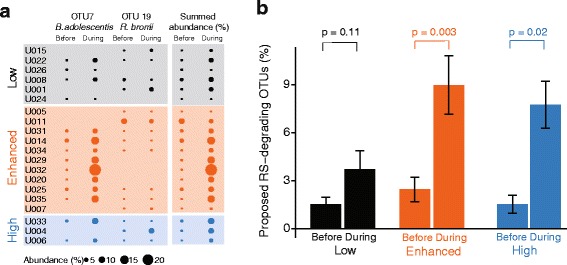
Identifying microbiota features that distinguish the low from other groups. **a** Median abundance of OTUs taxonomically related to *B. adolescentis* and *R. bromii* before and during RS for each individual. **b** Summed abundance of the two proposed RS-degrading OTUs before and during RS in the three groups

The OTUs that distinguished these three clusters were identified using Random Forest analysis and LEfSe (Table 3). Random Forest revealed OTU #7 as the most prominent feature of the microbiota distinguishing the low from the enhanced group (Table 3). Sequences within this OTU are identical to those from *Bifidobacterium adolescentis*. The relative abundance of this OTU before RS was similar in all three groups (~0.7–1.4 %; *t* test *p* > 0.27; Fig. 4a). However, the enhanced and high groups had dramatically higher abundances of this OTU during RS (average 8.9 % in enhanced and 7.8 % in high; *p* < 0.05; Fig. 4a). The abundance of this OTU did not change in the low group (average before RS = 1.5 %, average during = 3.7 %, *p* = 0.13). This finding was further corroborated with a LEfSe analyses comparing four samples before to four during RS consumption for each individual. As one would expect, the abundance of OTU 7 increased in 8 out of 11 individuals in the enhanced group, 2 out of 3 individuals in the high group, but in only 2 out of 6 individuals in the low group (LEfSe *p* < 0.05 correcting for multiple comparisons).

**Table 3:** Results of Random Forest regression and LEfSe to identify OTUs that distinguish the three response groups

Comparison	Distinguishing microbiota features	
	LEfSe	Random Forest
Low *vs.* enhanced	None identified	[OTU 7] _during RS_
High *vs.* low	[OTU 4] _before and during RS_	[OTU 4] _before and during RS_
		[OTU 3] _before RS_^‡^
High *vs.* enhanced	[OTU 4] _before and during RS_	[OTU 4] _before and during RS_

^‡^A post-hoc ANOVA analysis revealed that the abundance of OTU #3 was not significantly different between the groups being compared (*p* > 0.10). Hence, this OTU was not considered further

Cultivars of *B. adolescentis* are capable of breaking down RS [23]. OTU #7 in which sequences were identical to the 16S rRNA encoding gene of *B. adolescentis* was detected in 14 out of 20 total individuals and increased in abundance in 12 individuals. This OTU did not increase in individuals U026 and U024 (LEfSe *p* > 0.05 correcting for multiple comparisons). However, OTU #7 [*B. adolescentis*] was not detected in six individuals. In these individuals, LEfSe was used to find other OTUs that increased in abundance during RS supplementation, since these could be RS-degrading bacteria. In three individuals in whom *B. adolescentis* sequences did not increase, OTU 19 increased in abundance (LEfSe *p* < 0.05 correcting for multiple comparisons). Sequences in that OTU are identical to *Ruminococcus bromii*, another group of RS-degrading bacteria [23]. In individual U005, neither *B. adolescentis* nor *R. bromii* increased in abundance with RS. A potential candidate for a RS-degrading organism in this individual is OTU 50, whose average abundance increased from 2.6 to 7.1 % albeit not statistically significant (*p* = 0.29). Sequences in this OTU are most closely related to *Ruminiclostridium* [*Eubacterium*] *siraeum*. This organism has not been reported to degrade RS, but it is in the same taxonomic family as *R. bromii*.

Since *B. adolescentis* and *R. bromii* are the strongest candidates for RS-degrading organisms in this study, we summed the abundance of these two organisms for each individual (Fig. 4b). Individuals in all three groups start with a similar abundance of these RS-degrading microbes. RS elicits dramatic increases in their abundance in high and enhanced groups, but not in the low group (Fig. 4b).

**Fig. 5:**
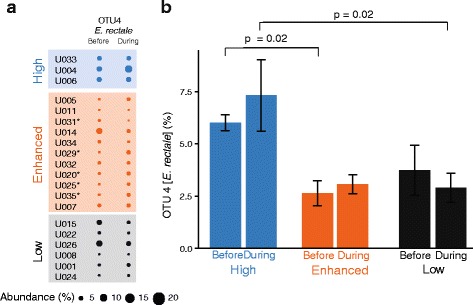
The relative abundance of OTU 4 *Eubacterium rectale* distinguishes the high from other groups. **a** Median abundance of OTU 4 before and during RS for each individual. Individuals in whom the abundance of this OTU increased with RS are marked with an asterisk (LEfSe *p* < 0.05). **b** Abundance of OTU 4 before and during RS in the three groups

Random Forest and LEfSe also revealed that OTU 4 distinguished the high group from the enhanced and low groups (Table 3). Sequences within this OTU are identical to *E. rectale*—a known and prominent butyrogenic microbe in human guts [24]. *E. rectale* was more abundant in individuals in the high group than in the other two groups—both before and during RS (Fig. 5b). While individuals in the high group fall into a very distinct cluster in the k-means clustering (Fig. 3), there are only three individuals in this group. This limited sample size does constrain the strength of the finding that *E. rectale* is more abundant in the high group *vs.* the other two groups. Some individuals in the enhanced group exhibited increases in abundances of *E. rectale* during RS supplementation (Fig. 5a, LEfSe *p* < 0.05 correcting for multiple comparisons), but in general, its abundance did not change as a result of RS addition to diet in any of the three groups (Fig. 5b). We looked specifically at other butyrogenic organisms known to be present in the human colon such as *Faecalibacterium prausnitzii* [25]*.* An OTU with the same V4 16S-rRNA encoding gene sequence as *F. prausnitzii* was present at about 3–6 % relative abundance in all three groups, but did not change with RS in any of the groups (*t* test *p* > 0.15). Since *B. adolescentis*—the prominent RS-degrading microbe in our cohort—produces lactate as the primary fermentation product, we also investigated if organisms known to produce butyrate from lactate increased in abundance. OTUs in which sequences were most closely related to *Eubacterium halii* and *Anaerostipes caccae* were present at less than 0.4 % abundance in our cohort and did not change with RS (paired *t* test *p* = 0.4).

## Discussion

The impact of dietary supplementation with resistant starch on fecal butyrate concentrations and the composition of the microbiota was examined in 20 healthy young adults. In this cohort, the average ratios of acetate:propionate:butyrate before and during RS supplementation were 58:25:15 and 58:22:20, respectively. These ratios agree well with previously documented ratios of ~60:20:20 in human feces [26–29]. In response to RS supplementation, the concentration of fecal butyrate increased from 8 to 12 mmol/kg wet feces in the overall study population (repeated measures ANOVA *p* = 0.02). The inter-individual variation in butyrate concentrations before and during RS was striking (Fig. 2). Most previous studies with RS have documented only population-wide response in butyrate concentrations [16], while one other study reported considerable heterogeneity between individuals in regard to fecal butyrate concentrations [30]. This pronounced inter-individual variability suggests that a single approach to improving beneficial functions from the microbiome is unlikely to be universally successful. Rather, personalized approaches may be needed to manage microbiomes for health. In line with this endeavor, we attempted to identify features in the microbiota that could explain different responses to dietary supplementation with RS.

Random Forest was used to identify relationships between butyrate concentrations and OTUs in the study population. Weak relationships were identified between butyrate concentrations before RS and the abundances of OTU 4 (*E. rectale*). Random Forest failed to reveal any relationships between OTU abundances and butyrate concentrations during RS supplementation. It is possible that the number of individuals in this study (*n* = 20) may have been too small to detect robust correlations between butyrate and the abundance of OTUs at the population level. Alternatively, it is not necessary that the abundance of a single organism be correlated with butyrate concentrations. After all, the potential for producing butyrate is fairly widespread within the phylum *Firmicutes* [31]. Rather, we expect that the abundance of genes encoding butyryl-CoA:acetate CoA transferase (*but*) and butyrate kinase (*buk*) would be correlated to butyrate concentrations.

Our next step was to create groupings in the data based upon similar responses to RS. This clustering approach should constrain the variability within each group and increase the probability of identifying microbiota features that characterize each type of response. K-means clustering using butyrate concentrations before and during RS identified three groups: enhanced, high, and low. Butyrate increased on average from 9 to 15 mmol/kg wet feces in the enhanced group, whereas butyrate concentrations remained consistently high or low in the other two groups (≥11 and ≤8 mmol/kg wet feces, respectively; Fig. 3). With these clusters, we were able to identify features of the microbial communities that differentiated the three groups.

The relative abundance of proposed RS-degrading bacteria—*B. adolescentis* or *R. bromii*—increased with RS supplementation in the enhanced and high groups (from 2 to 9 %), but not in the low group (~1.5 % throughout; Fig. 4). This finding complements and extends previously published reports on the effects of RS supplementation on the composition of the human gut microbiota. In a study with overweight adult males, only individuals with detectable abundances of *R. bromii* in their gut microbiota were able to degrade RS (type 3—Novelose 330; Walker et al. [32]). In another study with ten human subjects (five males and females; 28–38 years old), dietary supplementation with either type 4 RS (FiberSym RW) or type 2 RS (HiMaize260) increased the relative abundance of *B. adolescentis* and *R. bromii*, respectively (Martinez et al. [33]). These in vivo studies are nicely complemented by in vitro microcosm studies, which showed that isolates of *R. bromii* and *B. adolescentis* are capable of degrading several forms of RS [23]. Thus, different types of resistant starches are likely to promote the growth of *B. adolescentis* or *R. bromii*, with both organisms exhibiting substrate specificity depending upon the type and source of RS.

The relative abundance of RS-degrading organisms and butyrate concentrations did not increase in the low group. This suggests that their microbiota did not break down RS and so could not lead to increased butyrate production. This suggestion is supported by the fact that the concentrations of neither acetate nor propionate increased in the low group. So why does the abundance of RS-degrading organisms remain at 1.5 % in the low group? Possible explanations include limitation by antagonistic microbes or lack of synergistic microbes. Our attempts to identify antagonistic and synergistic interactions by constructing correlation networks [34] between RS-degrading organisms and other OTUs did not yield any compelling relationships.

In addition to RS-degrading organisms, another OTU (OTU #4) was identified as distinguishing the high group from the enhanced and low groups (Table 3, Fig. 5). Its V4 sequence is identical to that of *E. rectale*, well established as a prominent butyrogenic bacterium in the human gut microbiome [24, 35]. Its relative abundance in the high group was consistently about 6 %, compared to 3 % in the other two groups. Surprisingly, the abundance of *E. rectale* did not change in any of the groups in response to RS—even though butyrate concentrations increased appreciably in the enhanced group (from 9 to 15 mmol/kg wet feces). *E. rectale* generates butyrate from acetate, and there is a net gain of ATP in that process [35]. So if *E. rectale* was responsible for increased butyrate production in the enhanced group with RS, one might expect an increase in its relative abundance. We offer two possible explanations for the increased butyrate production in the enhanced group without a measurable increase in the abundance of *E. rectale*. First, changes in the relative abundance of *E. rectale* may be subtle and masked by the dramatic increase in the abundance of RS-degrading organisms (~from 2 to 10 % in the enhanced group). It is also possible that populations of *E. rectale* take longer to respond to RS supplementation. We used an acclimatization period of only 3 days during which the amounts of RS in diet were gradually increased, and after this period, four fecal samples were collected. It remains to be seen whether there is a detectable increase in the abundance of *E. rectale* following a longer duration of RS consumption. Also, as mentioned earlier, it is well documented that butyrate production is widespread within the phylum *Firmicutes* in human gut microbiomes. Therefore, rather than a single organism, if more acetate is being converted to butyrate, the genes encoding butyryl-CoA:acetate CoA transferase (*but*) and butyrate kinase (*buk*) should increase in abundance with RS supplementation.

## Conclusions

Our data show that dietary supplementation with RS type 2 as unmodified potato starch increases fecal butyrate concentration, but with remarkable inter-individual variation. We were able to infer potential explanations for some of these differential effects by investigating the composition of microbiota. Fecal butyrate concentrations increased by an average of 67 % in a subset of the study population (*n* = 11 of 20; 9 to 15 mmol/kg wet feces). Most individuals in this group showed a dramatic increase in the relative abundance of the RS-degrading organisms—*B. adolescentis* or *R. bromii*. In five of these individuals, the prominent butyrogenic microbe *E. rectale* also increased in abundance. Another subset of the population (3 of 20) consistently maintained high butyrate concentrations both before and with RS (≥12 mmol/kg wet feces). In this subset, RS-degrading organisms increased in abundance, suggesting that RS is being degraded. But there was no concomitant increase in fecal butyrate concentrations. These individuals may be experiencing a plateau effect [36] in butyrate production even before RS is administered. In fact, the microbiota structures of these individuals also did not change as much with RS (ANOSIM *R* = 0.17) as compared to changes in the microbiota in the enhanced group (ANOSIM *R* = 0.64). It is tempting to suggest that the microbiota of these individuals are performing well with regard to butyrate production, and they do not benefit from additional dietary input of fermentable carbohydrates. A third subset of our study population (6 of 20) had consistently low concentrations of butyrate even when consuming RS (≤8 mmol/kg wet feces). In this group, RS-degrading organisms did not increase in abundance, suggesting that their microbiota did not break down RS. Based upon this result, we propose that increasing butyrate in these individuals will require either (i) testing another form of dietary fiber such as inulin or arabinoxylan that their microbiota might degrade, (ii) a synbiotic approach that combines a dietary fiber with the appropriate fiber-degrading bacteria, or (iii) targeted removal of microbes if any are antagonistic to RS-degrading organisms. The findings of this study illustrate the importance of studying individual responses to dietary modifications. This will uncover the mechanisms that underlie these responses and in time provide actionable insights towards precision management of microbiomes.

## Abbreviations

ANOVA, analysis of variance; DNA, deoxyribose nucleic acid; g, gram(s); h, hour(s); HPLC, high-pressure liquid chromatography; LC, liquid chromatography; ml, milliliter(s); mM, millimolar; °C, degrees centigrade; OTU, operational taxonomic unit; rRNA, ribosomal RNA; RS, resistant starch; μl, microliters

## References

[1] Tremaroli V, Backhed F (2012). Functional interactions between the gut microbiota and host metabolism. Nature.

[2] Flint HJ, Duncan SH, Scott KP, Louis P (2015). Links between diet, gut microbiota composition and gut metabolism. Proc Nutr Soc.

[3] Hartstra AV, Bouter KE, Backhed F, Nieuwdorp M (2015). Insights into the role of the microbiome in obesity and type 2 diabetes. Diabetes Care.

[4] Zackular JP, Baxter NT, Iverson KD, Sadler WD, Petrosino JF, Chen GY, et al. The gut microbiome modulates colon tumorigenesis. mBio. 2013;4(6). doi:10.1128/mBio.00692-13. 10.1128/mBio.00692-13 PMC3892781 24194538

[5] Blanton LV, Charbonneau MR, Salih T, Barratt MJ, Venkatesh S, Ilkaveya O, et al. Gut bacteria that prevent growth impairments transmitted by microbiota from malnourished children. Science. 2016;351(6275):3311-1–7. 10.1126/science.aad3311 PMC4787260 26912898

[6] Buffie CG, Bucci V, Stein RR, McKenney PT, Ling L, Gobourne A (2015). Precision microbiome reconstitution restores bile acid mediated resistance to Clostridium difficile. Nature.

[7] Donohoe DR, Garge N, Zhang X, Sun W, O’Connell TM, Bunger MK (2011). The microbiome and butyrate regulate energy metabolism and autophagy in the mammalian colon. Cell Metab.

[8] Peng L, Li ZR, Green RS, Holzman IR, Lin J (2009). Butyrate enhances the intestinal barrier by facilitating tight junction assembly via activation of AMP-activated protein kinase in Caco-2 cell monolayers. J Nutr.

[9] Furusawa Y, Obata Y, Fukuda S, Endo TA, Nakato G, Takahashi D (2013). Commensal microbe-derived butyrate induces the differentiation of colonic regulatory T cells. Nature.

[10] Ruemmele FM, Schwartz S, Seidman EG, Dionne S, Levy E, Lentze MJ (2003). Butyrate induced Caco-2 cell apoptosis is mediated via the mitochondrial pathway. Gut.

[11] Mikkelsen KH, Allin KH, Knop FK (2016). Effect of antibiotics on gut microbiota, glucose metabolism and body weight regulation: a review of the literature. Diabetes Obes Metab.

[12] Mathewson ND, Jenq R, Mathew AV, Koenigsknecht M, Hanash A, Toubai T (2016). Gut microbiome-derived metabolites modulate intestinal epithelial cell damage and mitigate graft-versus-host disease. Nat Immunol.

[13] Mathewson N, Mathew A, Oravecz-Wilson K, Wu J, Toubai T, Cummings E (2014). Microbial metabolites modulate GI mucosal damage from graft versus host disease (GHVD). (MUC4P.850). J Immunol.

[14] Smith MI, Yatsunenko T, Manary MJ, Trehan I, Mkakosya R, Cheng J (2013). Gut microbiomes of Malawian twin pairs discordant for kwashiorkor. Science.

[15] Ohland Christina L, Jobin C (2014). Bugs and food: a recipe for cancer?. Cell Metab.

[16] Birt DF, Boylston T, Hendrich S, Jane J-L, Hollis J, Li L (2013). Resistant starch: promise for improving human health. Adv. Nutr Int Review J.

[17] Kozich JJ, Westcott SL, Baxter NT, Highlander SK, Schloss PD (2013). Development of a dual-index sequencing strategy and curation pipeline for analyzing amplicon sequence data on the MiSeq Illumina sequencing platform. Appl Environ Microbiol.

[18] Schloss PD, Westcott SL, Ryabin T, Hall JR, Hartmann M, Hollister EB (2009). Introducing mothur: open-source, platform-independent, community-supported software for describing and comparing microbial communities. Appl Environ Microbiol.

[19] R Development Core Team. R: a language and environment for statistical computing. In: Computing RFfS, editor. Vienna, Austria: R Foundation for Statistical Computing; 2015.

[20] Hammer O, Harper DAT, Ryan PD. PAST: paleontological statistics software package for education and data analysis. In: Electronica P, editor.2001.

[21] Segata N, Izard J, Waldron L, Gevers D, Miropolsky L, Garrett WS (2011). Metagenomic biomarker discovery and explanation. Genome Biol.

[22] Thorndike RL. Who belongs in the family? Psychometrika.18(4):267-76. doi:10.1007/bf02289263.

[23] Ze X, Duncan SH, Louis P, Flint HJ (2012). *Ruminococcus bromii* is a keystone species for the degradation of resistant starch in the human colon. ISME J.

[24] Duncan SH, Flint HJ (2008). Proposal of a neotype strain (A1-86) for *Eubacterium rectale*. Request for an opinion. Int J Syst Evol Microbiol.

[25] Khan MT, Duncan SH, Stams AJM, van Dijl JM, Flint HJ, Harmsen HJM. The gut anaerobe *Faecalibacterium prausnitzii* uses an extracellular electron shuttle to grow at oxic-anoxic interphases. ISME J. 2012:doi:10.1038/ismej.2012.5. 10.1038/ismej.2012.5 PMC3400418 22357539

[26] den Besten G, van Eunen K, Groen AK, Venema K, Reijngoud D-J, Bakker BM (2013). The role of short-chain fatty acids in the interplay between diet, gut microbiota, and host energy metabolism. J Lipid Res.

[27] Hijova E, Chmelarova A (2007). Short chain fatty acids and colonic health. Bratisl Lek Listy.

[28] Binder HJ (2010). Role of colonic short-chain fatty acid transport in diarrhea. Annu Rev Physiol.

[29] Cummings JH, Pomare EW, Branch WJ, Naylor CP, Macfarlane GT (1987). Short chain fatty acids in human large intestine, portal, hepatic and venous blood. Gut.

[30] McOrist AL, Miller RB, Bird AR, Keogh JB, Noakes M, Topping DL (2011). Fecal butyrate levels vary widely among individuals but are usually increased by a diet high in resistant starch. J Nutr.

[31] Vital M, Howe AC, Tiedje JM. Revealing the bacterial butyrate synthesis pathways by analyzing (meta)genomic data. mBio. 2014;5(2). doi:10.1128/mBio.00889-14. 10.1128/mBio.00889-14 PMC3994512 24757212

[32] Walker AW, Ince J, Duncan SH, Webster LM, Holtrop G, Ze X, et al. Dominant and diet-responsive groups of bacteria within the human colonic microbiota. ISME J. 2011;5(2):220–30. doi:10.1038/ismej.2010.118. PubMed PMID: PMC3105703. 10.1038/ismej.2010.118 PMC3105703 20686513

[33] MartÌnez I, Kim J, Duffy PR, Schlegel VL, Walter J (2010). Resistant starches types 2 and 4 have differential effects on the composition of the fecal microbiota in human subjects. PLoS One.

[34] Friedman J, Alm EJ (2012). Inferring correlation networks from genomic survey data. PLoS Comput Biol.

[35] Rivière A, Gagnon M, Weckx S, Roy D, De Vuyst L (2015). Mutual cross-feeding interactions between *Bifidobacterium longum* subsp. longum NCC2705 and *Eubacterium rectale* ATCC 33656 explain the bifidogenic and butyrogenic effects of arabinoxylan oligosaccharides. Appl Environ Microbiol.

[36] Guggenheim KY (1991). Rudolf Schoenheimer and the concept of the dynamic state of body constituents. J Nutr.

